# Osteoma of the middle ear

**DOI:** 10.1016/S1808-8694(15)30137-3

**Published:** 2015-10-19

**Authors:** Vinicius Cotta Barbosa, Marco Aurélio Rocha Santos, Helena Maria Gonçalves Becker, Renata Furletti Diniz

**Affiliations:** a Otorhinolaryngologist of the Mater Dei Hospital; b PhD in Sciences - Federal University of São Paulo - UNIFESP Post-Graduate Program. Otorhinolaryngologist of the University Hospital - Federal University of Minas Gerais; c PhD in Medicine - Federal University of Minas Gerais; Adjunct Professor of the Otorhinolaryngology, Ophthalmology and Speech and Hearing Therapy Department - Federal University of Minas Gerais; d MD. Radiologist - Mater Dei Hospital. Serviço de Otorrinolaringologia do Hospital das Clínicas da Faculdade de Medicina da Universidade Federal de Minas Gerais

**Keywords:** middle ear, osteoma, tomography

## INTRODUCTION

Osteomas are rare benign bone tumors of the bone lamellar portion, that usually originate from the paranasal sinuses where they are pedicled. In the temporal bone, they are usually found in the external auditory canal and are extremely rare in the middle ear. Temporal bone osteomas have a preference for the outermost portion of the external auditory canal, while exostoses affect its most internal portion. In the temporal bone, its presence has been described in the mastoid, in the squama and in the internal auditory canal, however with marked preference for the external auditory canal1. In the medical literature, there are 60 cases of temporal bone osteomas described, originating outside the external acoustic meatus2. According to Harley et al.3 only 12 of these osteomas were found in the middle ear; and they also reported that only five of these patients complained of hearing loss, while the others were diagnosed by chance. Unal et al.4 also spoke about the conduction hearing loss in cases of middle ear osteomas. Silver et al.5 are among the authors that stressed the lack of symptoms in middle ear osteomas and, on the other hand, Muraoka et al.6 showed how important CT scan is in otorhinolaryngology in general, most specially the 3D type. Hearing loss is conductive, unilateral and progressive. Temporal bone osteomas occur mostly in young patients, present as single-unilateral lesion, of unknown etiology. It is possible that they originate from the bone capsule connective tissue and they are histologically similar to those observed in the external acoustic canal. The goal of this paper is to report a case of middle ear osteoma associated with conductive hearing loss, for which the diagnosis hypothesis was of otosclerosis.

## CASE PRESENTATION

**Figure 1:**
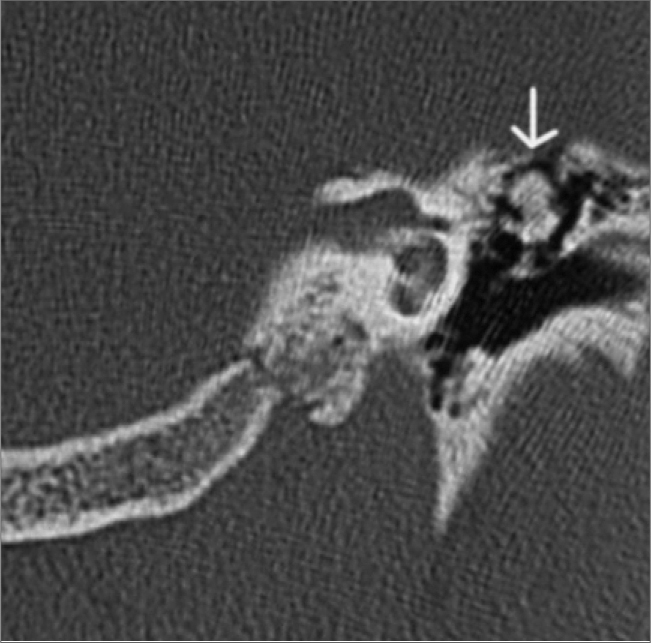
Mastoid coronal view CT scan, showing the osteoma.

D.C.S., 23 year old male student, born in Belo Horizonte, came to us complaining of progressive left side hearing loss associated with a mild tinnitus in the previous 3 months, with no family history of hearing loss. Otoscopic examination did not reveal ear drum or middle ear alteration in any of the sides. Tuning fork tests confirmed left side hearing loss; Weber’s test lateralized to the left side and negative Rinne’s test. Audiogram confirmed a moderate conductive hearing loss and impedanciometry showed an Ar curve. A temporal bone CT scan without contrast was ordered in axial and coronal views, which showed a calcic density image on the left epitympanum, contiguous with the incus short arm, measuring 0.6 x 0.8 mm, with well defined boundaries, going from the Chaussé spur, all the way to the lateral semicircular canal. The stapes was free and there now joint alterations between the malleous and the incus (Figure 1).

## DISCUSSION

Middle ear osteomas affect mainly the young population, it may cause conductive-type progressive hearing loss and bear normal otoscopic exam. Although it is a rare type of disorder, one must be attentive towards such possibility; and it is important to consider the differential diagnosis with other middle ear disorders, especially fenestra otospongiosis, because of its higher prevalence. The temporal bone CT scan, besides providing additional information on the Fallopian Canal route, its relationship with the stapes, the status of the other ossicula, it provides for an assessment of pre and post fenestra otosclerosis, considering clinical manifestations such as those described here allows for less operative surprises and more accurate diagnosis.

## FINAL COMMENTS

For most of the authors in the literature, the middle ear osteoma treatment is based on observation until the time when the hearing loss becomes relevant for the patient or, when there are other complications related. This was the treatment offered to and accepted by the patient. The patient was also advised that in his case, surgery would approach the incus and the stapes, different from the previous one that involved the stapes and the footplate when considering otosclerosis. Thus, together with the patient, we decided for a follow up of his hearing loss with audiometric and tomographic exams.

Although rare, osteomas should be considered in the differential diagnosis of conductive hearing loss. Temporal bone CT scan, in cases of fenestra otospongiosis, provides additional information on the oval window, the stapes, the cochlea, the Fallopian canal and the other ossicula, which helps to check for other alterations which were not clinically suspected.

## References

[1] Ramadam HH (1994). Osteoma of the malleus. Am J Otol.

[2] Tanaka H, Ito S, Hirano M (1990). Osteoma of the middle ear. J Laryngol Otol.

[3] Harley EH, Berkowitz RG (1997). Osteoma of the middle ear. Ann Otol Rhinol Laryngol.

[4] Unal OF, Tosun F, Yetser S, Dundar A (2000). Osteoma of the middle ear. International J Pediatr Otorhinolaryngol.

[5] Silver FM, Orobello PW, Mangal A, Pensak ML (1993). Asymptomatic oteomas of the midlle ear. Am J Otol.

[6] Muraoka A, Tsunoda A, Kojima M, Tokano H, Komatsuzaki A (1996). Three dimensional imaging of ENT diseases with helical computed tomography. J Laryngol Otol.

